# Development of a Bispecific IgG1 Antibody Targeting BCMA and PDL1

**DOI:** 10.3390/antib13010015

**Published:** 2024-02-20

**Authors:** Irene Cattaneo, Sylvie Choblet, Rut Valgardsdottir, Muriel Roth, Annamaria Massafra, Marten Beeg, Marco Gobbi, Martine Duonor-Cerutti, Josée Golay

**Affiliations:** 1 Division of Hematology, Center of Cellular Therapy “G. Lanzani”, Azienda Socio Sanitaria Territoriale Papa Giovanni XXIII, 24122 Bergamo, Italy; icattaneo@asst-pg23.it (I.C.); rutvalg@gmail.com (R.V.); 2 Centre National de la Recherche Scientifique UAR3426 “Baculovirus et Therapie”, 30380 Saint-Christol-Lez-Alès, France; sylvie.choblet@cnrs.fr (S.C.); muriel.roth@cnrs.fr (M.R.); martine.cerutti@cnrs.fr (M.D.-C.); 3 Laboratory of Pharmacodynamics and Pharmacokinetics, Department of Biochemistry and Pharmacology, Istituto di Ricerche Farmacologiche Mario Negri—IRCCS, 20157 Milan, Italy; annamaria.massafra@unimore.it (A.M.); marten.beeg@marionegri.it (M.B.); marco.gobbi@marionegri.it (M.G.)

**Keywords:** bispecific antibody, multiple myeloma, BCMA, checkpoint inhibitor, PDL1

## Abstract

We designed, produced, and purified a novel IgG1-like, bispecific antibody (bsAb) directed against B-cell maturation antigen (BCMA), expressed by multiple myeloma (MM) cells, and an immune checkpoint inhibitor (ICI), PDL1, expressed in the MM microenvironment. The BCMA×PDL1 bsAb was fully characterized in vitro. BCMA×PDL1 bound specifically and simultaneously, with nM affinity, to both native membrane-bound antigens and to the recombinant soluble antigen fragments, as shown by immunophenotyping analyses and surface plasmon resonance (SPR), respectively. The binding affinity of bsAb for PDL1 and BCMA was similar to each other, but PDL1 affinity was about 10-fold lower in the bsAb compared to parent mAb, probably due to the steric hindrance associated with the more internal anti-PDL1 Fab. The bsAb was also able to functionally block both antigen targets with IC_50_ in the nM range. The bsAb Fc was functional, inducing human-complement-dependent cytotoxicity as well as ADCC by NK cells in 24 h killing assays. Finally, BCMA×PDL1 was effective in 7-day killing assays with peripheral blood mononuclear cells as effectors, inducing up to 75% of target MM cell line killing at a physiologically attainable, 6 nM, concentration. These data provide the necessary basis for future optimization and in vivo testing of this novel bsAb.

## 1. Introduction

Although a number of non-conjugated monoclonal antibodies (mAbs) have demonstrated significant therapeutic activity against cancer, many others have shown poor efficacy or unacceptable toxicity [1,2,3]. Bispecific antibodies (bsAbs) allow the targeting of two different antigens, thus adding another function to the drug compared to mAbs. BsAbs can also be trifunctional, for example, if they carry a fully active human IgG1 Fc capable of binding FcɣRs and activating immune cells. Fc also binds to FcRn and prolongs antibody half-life in vivo through recycling [4]. Thus, the choice of specificities and overall structure of novel bsAbs has to consider the final biological activities that are desired from the drug. Relative affinities of the two arms of a bsAb are also important elements to consider during development, according to the desired function of the molecule and properties of the antigens that are targeted [5].

We have recently developed a novel platform for bsAb engineering, which allows us to produce IgG-like, Fc-bearing bsAbs with bivalent binding to each chosen antigen [6]. Using this platform, we have set out to design a novel bispecific molecule to target multiple myeloma (MM). We have focused our attention on B-cell maturation antigen (BCMA) as the primary specificity, because this molecule is expressed by mature normal and neoplastic B cells, including MM, plasma cell leukemia (PCL), as well as some differentiated diffuse large B-cell lymphomas (DLBCLs), but is mostly absent from other tissues [7,8]. However, membrane BCMA (mBCMA) is expressed at relatively low levels on MM and DLBCL cells, in part due to shedding of its extracellular domain by membrane-associated ɣ-secretase, so unconjugated anti-BCMA mAbs also have poor efficacy in vivo [9,10]. Different approaches have been applied to develop more effective anti-BCMA therapeutics, in particular T cell-engaging bispecifics, antibody–drug conjugates, and CAR-T cells, each of which may have advantages and limitations [11,12,13].

Immune checkpoint inhibitors (ICIs) are expressed by tumor cells or their microenvironment and diminish the activity of anti-cancer drugs, including immunotherapeutics. In particular, MM and DLBCL cells or tissues often express programmed cell death protein 1 (PD1) and/or its ligand programmed death-ligand 1 (PDL1). We reasoned that adding an anti-PDL1 moiety to an anti-BCMA antibody in a bispecific format may thus allow the PD1/PDL1 axis to be blocked within the tumor, since the anti-BCMA moiety would concentrate the antibody mostly in the tumor bed, potentially increasing the efficacy of the anti-BCMA and reducing the systemic toxicity of the anti-PDL1 moiety. A BCMA×PDL1 bsAb was thus designed and produced, based on the IgG1 format developed and patented in our laboratory [6]. In this report, we describe the construction of this novel tetravalent Fc-bearing bsAb and characterize its antigen binding and functional activities in vitro.

## 2. Materials and Methods

### 2.1. Therapeutic Antibodies

The construction of the transfer vectors carrying light chains and fused or standard chimeric IgG1 heavy chains for bsAb and mAb was performed by genetic engineering, essentially as described previously (patent WO/2013/005194) [6,14]. The antibodies were based on the VH/VL sequences of anti-BCMA J22.9 antibody (patent WO 2014/068079) [15] and anti-PDL1 atezolizumab (MPDL3280A, US8217149B2). The generation and cloning of recombinant baculoviruses expressing 2 chains (mAbs) or 3 chains (bsAb), antibody expression in insect *Sf9* cells, and purification were performed as described [6,14,16].

The following antibodies were investigated, anti-BCMA, anti-PDL1, and anti-BCMA×PDL1, all produced in *Sf9* cells and purified at CNRS. In some experiments, commercially available anti-PDL1 atezolizumab (Tecentriq^®^, Roche, Basel, Switzerland) was also used as control. IgG mix from human serum (Sigma-Aldrich, St. Louis, MO, USA) was used as reference for SPR analysis. Anti-CD38 daratumomab (DARA), anti-CD20 rituximab (RTX), anti-EGFR cetuximab, and commercial atezolizumab, used either as positive or negative controls, were obtained from the local pharmacy.

### 2.2. Cells

Peripheral blood (PB) or bone marrow (BM) samples were collected in EDTA from healthy donors or patients diagnosed with either MM or B-cell non-Hodgkin-lymphoma (B-NHL). This study was approved by local ethical committee and samples were collected after informed consent and in accordance with the Declaration of Helsinki of 1975, as amended in 2013. Mononuclear cells (MNCs) were purified by standard Ficoll-Hypaque gradient centrifugation (Seromed, Biochrom AG, Berlin, Germany). In some cases, MM cells from BM were first purified on anti-CD138 immunoaffinity columns (AutoMACS, Miltenyi Biotec, Bergisch Gladbach, Germany). Cells were either used immediately or cryopreserved in 10% DMSO (Li StarFish, Milan, Italy) for later use.

The human cell lines used in the present study were as follows: CEM (acute lymphoblastic leukemia, ALL); HDLM2 (Hodgkin lymphoma, HL); MEC-1 (chronic B leukemia, B-CLL); REH, TOM-1, and 697 (precursor B cell acute lymphoblastic leukemia, pre-B ALL); GRANTA 519 and JEKO-1 (mantle cell lymphoma, MCL); KARPAS422, DOHH2, SU-DHL4, SU-DHL16, and RCK8 (DLBCL); ALBANES, BJAB, RAMOS, RAJI, and NAMALWA (Burkitt lymphoma, BL); PA698 and AS238 (AIDS-derived non-Hodgkin B-cell lymphoma, AIDS-derived B-NHL); EBV-LCL from a healthy donor, IM9, and SKW6.4 (Epstein–Barr virus immortalized lymphoblastoid B-cell lines, EBV-LCL); and KMS11, KMS12, KMS18, KMS20, H929, OPM2, RPMI8226, and U266 (MM). All cell lines were maintained in RPMI-1640 medium (Life Technologies, Carlsbad, CA, USA) supplemented with 10% fetal bovine serum (FBS, Euroclone, Milan, Italy) and 2 mmol/L L-glutamine (Euroclone), except HDLM2, TOM-1, DHL4, and RAJI, which were supplemented with 20% FBS. Cell lines were routinely tested for the absence of mycoplasma.

### 2.3. Production of Stable BCMA Transfectants

BJAB cells were stably transfected with GFP-tagged full-length BCMA cDNA by infection with the BCMA-Lenti ORF-Myc/DDK/GFP-tagged lentivirus (Origene, Herford, Germany), following the manufacturer’s instructions. The BCMA-Lenti ORF encodes a puromycin antibiotic resistance gene for selection in mammalian cells. Stably infected cells were selected by supplementing the complete medium with 0.5 µg/mL of puromycin antibiotic, until >95% of BJAB-mBCMA^+^-expressing cells was reached. mBCMA expression was verified at various times and prior to experiments, by assessing the percentage of GFP and/or mBCMA-positive cells by flow cytometry (see below).

CEM cells (1.5 × 10^6^) were stably transfected with an expression plasmid bearing the full-length human BCMA cDNA (3 μg pUNO1-TNFRS17, InvivoGen, San Diego, CA, USA), using the nucleofector kit V and Amaxa IIb nucleofector device (Lonza, Basel, Switzerland). After 15 days of expansion, transfected CEM-mBCMA^+^ cells were purified by staining with a mouse anti-human BCMA antibody (Biolegend, San Diego, CA, USA) followed by FITC-labeled goat anti-mouse IgG secondary antibody (BD Biosciences, San Jose, CA, USA), and immunoselection was carried out with anti-FITC magnetic beads (Miltenyi Biotec). After two rounds of immunoselection, a purity of >94% of CEM cells stably expressing mBCMA was obtained. Expression was stable over time.

### 2.4. Flow Cytometry

mBCMA expression on cell lines was analyzed after overnight cell culture in the presence or absence of 1 µM ɣ-secretase inhibitor N-[N-(3,5-Difluorophenacetyl)-L-alanyl]-S-phenylglycine t-butyl ester (DAPT, Selleckchem, Cologne, Germany). Detection was performed by direct immunofluorescence and flow cytometry, using an anti-human BCMA-PE (clone 19F2, Biolegend) antibody.

The binding affinity of home-produced mAbs and bsAbs was measured by indirect immunofluorescence. The KMS11 (mBCMA^+^) or HDLM2 (PDL1^+^) cell lines were incubated with increasing equimolar concentrations of bsAb or mAbs, washed, and labeled with FITC-labeled anti-human Fc mAb (clone HP-6017, Sigma-Aldrich). Binding was measured by flow cytometry on a FACSCanto II instrument (BD Biosciences).

Either BM-derived MNCs (BM-MNC) or CD138^+^ purified cells were used for immunophenotypic analysis of primary MM. Peripheral blood mononuclear cells (PBMCs) were used in the case of B-NHL. Primary cells were cultured overnight in StemSpan SFEM medium (Stemcell Technologies, Vancouver, Canada), supplemented with 10% FBS in the presence or absence of 1 µM DAPT. After incubation, cells were stained with a mouse anti-human BCMA-PE-CY7 (clone 19F2, Biolegend) and mouse anti-human CD138-FITC (clone MI15), CD38-PerCp (clone HIT2) and CD19-APCH7 (clone HIB1) (BD Biosciences) (for MM cells), or mouse anti-human CD19-APCH7 and CD20-V450 (clone L27) (BD Biosciences) (for B-NHL). In some cases, BM-MNC and PBMC were also characterized for the expression of PD1 and PDL1 on neoplastic cells, T-lymphocytes, and monocytes by direct staining with mouse anti-human PD1-APC (clone MIH4), PDL1-PE (clone MIH1), CD3-APCH7 (clone SK7), and CD14-PerCp (clone MφP9) antibodies (BD Biosciences). For all antibodies, respective isotype controls were used. Antibodies were used at the concentrations suggested by the manufacturer. Stained cells were analyzed on a FACSCantoII cytometer.

### 2.5. Surface Plasmon Resonance (SPR)

SPR analysis was carried out with the ProteOn XPR36 Protein Interaction Array system (BioRad, Milan, Italy). The system allows up to six ligands to be immobilized on parallel lanes of the same sensor surface (including an “empty” or reference channel). The flow channels can be rotated 90° so that up to six analyte solutions can be flowed in parallel on all the immobilized ligands [17].

mAbs and bsAb were immobilized on the surface of a GLH CMD 700L sensor chip (XanTec bioanalytics, Düsseldorf, Germany) through an amine-coupling process, as previously described [18]. Briefly, the chip surface was activated by flowing a solution made by 50 mM N-hydroxysuccinimide and 400 mM 1-ethyl-3-(3-dimethylaminopropyl)-carbodiimide (NHS/EDC), for 5 min at 30 µL/min. The antibodies were then flowed for 5 min at 30 µL/min, at a concentration of 30 µg/mL in sodium-acetate pH 5.0 (NaOAc). The remaining activated carboxyl groups were deactivated by flowing 1 M ethanolamine for 5 min at 30 µL/min. Immobilization levels were 1359, 2400, and 4120 resonance units (RUs, where 1000 RU = 1 ng protein/mm^2^), for anti-BCMA, anti-PDL1, and BCMA×PDL-1 bsAb, respectively. A reference surface was prepared in a parallel channel of the same chip flowing commercial, unrelated IgGs, which immobilized at a level of 2352 RU.

After chip rotation, the following analytes, diluted in SPR running buffer (Dulbecco’s Phosphate-Buffered Saline with 0.005% Tween-20), were injected simultaneously on all the immobilized antibodies: recombinant human BCMA extracellular domain Fc chimera (recBCMA, R&D system, Minneapolis, MN, USA) and recombinant human PDL1 extracellular domain-Fc chimera (recPDL1, R&D system). Analytes flowed over immobilized ligands for 3 min at a rate of 30 μL/min. Dissociation was measured in the following 5–25 min. The SPR signals on the sensorgrams, expressed as RU, were corrected by subtracting the nonspecific response in the reference channel. The kinetic parameters, association and dissociation rate constants (k_a_ and k_d_), and the equilibrium dissociation constant (K_D_) were obtained by globally fitting the Langmuir model to entire sensorgrams (association and dissociation phases).

### 2.6. Inhibition of Binding of APRIL to mBCMA

CEM-mBCMA^+^ cells (2 × 10^5^) were incubated in the presence or absence of equimolar doses of BCMA×PDL1 bsAb or BCMA mAb (2.2 nM to 60 nM) for 10 min at 4 °C. After washing, cells were incubated for 20 min at 4 °C with 90 nM recombinant multimeric human Flag-tagged proliferation-inducing ligand (APRIL) protein (Adipogen Life Sciences, San Diego, CA, USA), followed by FITC-labeled mouse anti-Flag antibody (Sigma-Aldrich). Analysis was performed by flow cytometry on a FACSCanto II instrument.

### 2.7. Inhibition of PD1-PDL1 Signalling

To assess PDL1 functional blocking by anti-PDL1 antibodies, the cell-based PD1/PDL1 Blockade Bioassay (Promega, Madison, WI, USA) was used, according to the manufacturers’ instructions. Briefly, one day before performing the assay, the PDL1^+^ aAPC/CHO-K1 cells were seeded in a 96-well plate. The next day, PDL1^+^ aAPC/CHO-K target cells were incubated with serial 2.5-fold equimolar dilutions of PDL1 mAbs and BCMA×PDL1 bsAb or negative control cetuximab. The PD1^+^ Jurkat T effector cells were then added and the plate was incubated for 6 h in a 37 °C, 5% CO_2_ incubator. The bioluminescent signal was then activated with the Bio-Glo™ Luciferase Reagent and quantified on an FLUOstar OPTIMA plate reader (BMG Labtech GmbH, Ortenberg, Germany). In some experiments, the recBCMA (33 nM) or control recPDL1 were added to the blocking antibodies.

### 2.8. Complement-Dependent Cytotoxicity (CDC)

BJAB-mBCMA^+^ cells were incubated overnight with 1 µM of DAPT, and then plated at 2 × 10^5^ cells/well in a 96-well plate and exposed to equimolar concentrations of BCMA×PDL1 bsAb or mAbs, in the presence of 50% pooled human serum (HS), as a source of complement. RTX was used as positive control. After 4 h of incubation at 37 °C, the lysis was quantified by flow cytometry (FACSCanto II instrument) as percentage of 7-aminoactinomycin D (7AAD, BD Bioscience)-positive cells [14].

### 2.9. NK-Mediated ADCC (24 h Killing Assays)

The KMS11, OPM2, and KMS12 target cell lines were first cultured overnight with 1 µM DAPT, stained with 0.5 µM 5(6)-Carboxyfluorescein diacetate N-succinimidyl ester (CFSE; Sigma-Aldrich, Merck KGaA), and finally co-cultured with effector PBMCs, at a 5:1 E:T ratio, in the presence or absence of an equimolar concentration of BCMA×PDL1 bsAb or mAbs. DARA was used as positive controls. After 24 h [19], cells were harvested and stained using the GFP-certified TM Apoptosis/Necrosis detection kit (Enzo Life Science, Farmingdale, NY, USA), according to the manufacturer’s instructions. Samples were analyzed by flow cytometry (FACSCanto II instrument). Cell mortality is expressed as % Cytotoxicity calculated with the formula:

$$\%\;\mathrm{Cytotoxicity}=\frac{\%\;of\;death\;{CFSE}^{+}cells-\;\%\;spontaneous\;death\;{of\;CFSE}^{+}cells}{100-\%\;spontaneous\;death\;{of\;CFSE}^{+}cells}\times 100$$

In some cases, NK cell activation was measured after 4 h of co-culture of PBMC and KMS11 cells at a 5:1 E:T ratio, by staining with anti-human CD56-APC (BD Biosciences) and anti-human CD107a-PE (BD Biosciences) mAbs [14]. The degranulation of NK cells was quantified by flow cytometry (FACSCanto II instrument) as an increase in percentage of CD107a^+^ cells in the CD56^+^ population.

### 2.10. Long-Term Cell-Mediated Cytotoxicity (7-Day Killing Assays)

DAPT-treated KMS11 target cells were cocultured with effector PBMC, at a 5:1 E:T ratio, and 6 nM bsAb or mAbs in StemSpan SFEM medium supplemented with 10% FBS. After 3 days of culture, cells were split at a 1:2 ratio in fresh complete culture medium containing the same bsAb or mAbs. After 7 days [19,20], cells were collected and stained with 7-AAD and anti-human CD138-APCH-7 mAb (BD Biosciences). CountBright™absolute counting beads (Thermo Fisher, Waltham, MA, USA) were added to the staining tube. Absolute count of live 7-AAD-negative CD138^+^ KMS11 target cells was analyzed by flow cytometry, after gating for beads and for live cells based on forward and side scatter (FACSCanto II instrument).

### 2.11. Statistical Analyses

Statistical significance was calculated using Student’s *t*-test or Linear Regression *t*-test.

## 3. Results

### 3.1. mBCMA and PD1/PDL1 Expression in Neoplastic B Cells

**Figure 1:**
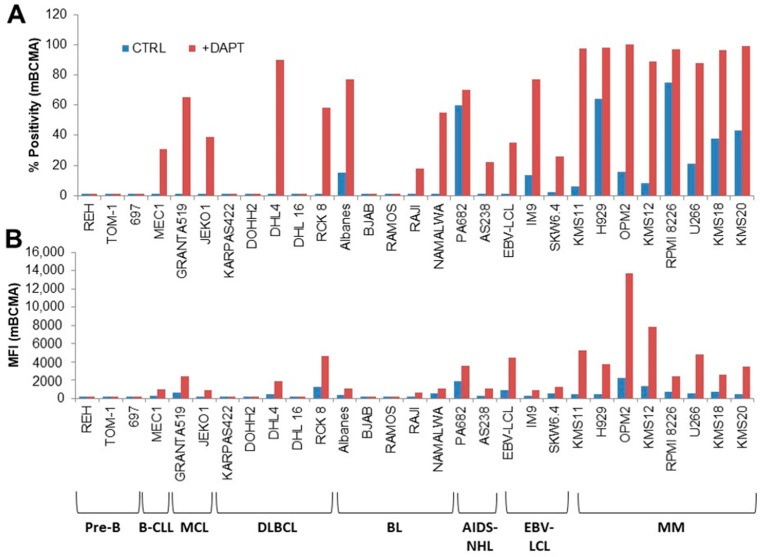
Expression of mBCMA in B cell lines. mBCMA expression was assessed by flow cytometry in B cell lines cultured in the presence or absence of ɣ-secretase inhibitor DAPT. (**A**) Percentage of mBCMA^+^ cells. (**B**) Mean fluorescence intensity, MFI. Pre-B ALL: precursor B cell acute lymphoblastic leukemia; B-CLL: chronic B leukemia; MCL: mantle cell lymphoma; DLBCL: diffuse large B-cell lymphoma; BL: Burkitt lymphoma; AIDS-NHL: AIDS-derived non-Hodgkin B-cell lymphoma; EBV-LCL: Epstein–Barr virus immortalized lymphoblastoid B-cell lines; MM: multiple myeloma.

mBCMA is known to be expressed on the surface of most MM cells and normal plasma cells (PCs). Some reports have detected mBCMA expression also on mature lymphoma cells, but this has been less systematically studied [8,21,22]. The mBCMA extracellular domain is shed from the cell surface by ɣ-secretase, with the rest of the molecule being rapidly degraded, resulting in a relatively weak basal expression of mBCMA on most MM cell lines and primary samples [9]. Blocking ɣ-secretase with inhibitors results in a 3- to 4-fold increase in mBCMA mean fluorescence intensity (MFI) [7,9,10]. We therefore investigated using flow cytometry the mBCMA expression in the presence or absence of ɣ-secretase inhibitor DAPT in a panel of B cell lines, from immature pre-B to fully differentiated MM cells. The data confirm the weak basal expression of mBCMA in all eight MM cell lines tested and the strong upregulation in the presence of DAPT (Figure 1).

Among the 21 other B cell lines analyzed, we observed that, in the absence of DAPT, mBCMA was low or negative in most cell lines, except for one AIDS-derived B-NHL (PA682, 60% positive, Figure 1A). In contrast, DAPT treatment resulted in >20% mBCMA surface expression in 12 B-NHL and EBV-LCL lines (ranging from 20 to 90% positivity). All mBCMA^+^-positive lines were more mature lymphoma cells: 2/2 MCL, 1 B-CLL, 2/5 DLBCL, 2/5 BL, 2/2 AIDS-derived B-NHL, and 3/3 EBV-LCL (Figure 1A). MFI analysis showed that three B-NHL cell lines, in the presence of DAPT, expressed mBCMA at an intensity similar to that of MM cell lines (Figure 1B).

We then analyzed using flow cytometry the primary samples from MM and B-NHL patients. As already described by others and by our group [7,9], most MM cells were positive for mBCMA but at a relatively low density, which was significantly and consistently increased by DAPT treatment (Supplementary Figure S1). The two primary Mantle Zone Lymphoma (MZL) samples also revealed a low density of mBCMA expression, which was induced by ɣ-secretase inhibitor (Supplementary Figure S1, patient numbers 22 and 23). These data confirm that MM and a proportion of more differentiated B-NHL cells may be targeted by anti-BCMA drugs, at least in conditions that block ɣ-secretase activity [8,21,22].

**Figure 2:**
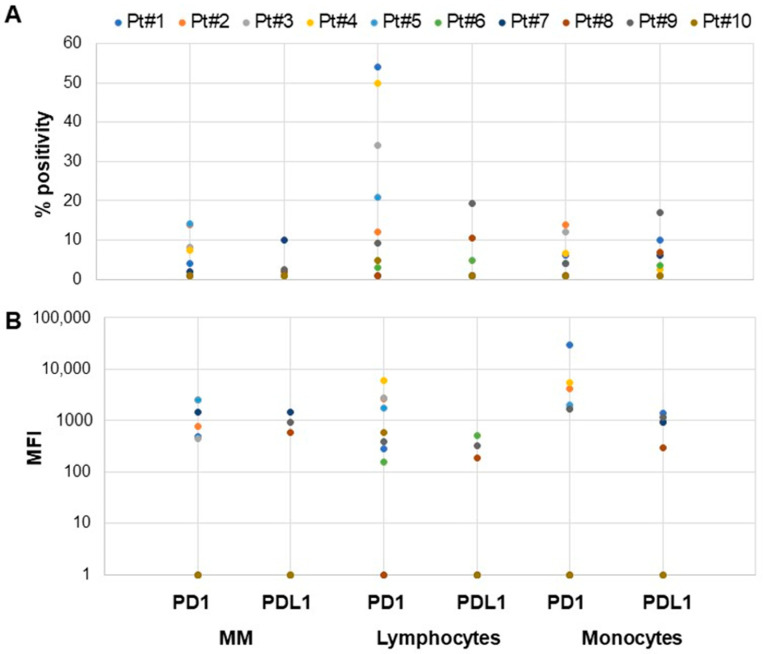
Expression of PD1 and PDL1 in BM samples from MM patients. Expression of PD1 and PDL1 in MM cells, T-lymphocytes, and monocytes was analyzed in 10 multiple myeloma BM samples. (**A**) Percentage of positive cells. (**B**) Mean fluorescence intensity, MFI.

We next investigated PD1 and PDL1 expression in MM and other cells present in the BM microenvironment. We could access 10 BM samples from MM patients. The data show that PD1/PDL1 expression was very variable between samples, with only 4/10 MM samples expressing either PD1 or PDL1 on >5% of cells (Figure 2A,B). However, T-lymphocytes and/or monocytes/macrophages present in the BM of MM patients did express either PD1 or PDL1 to a significant degree in 70% of cases (Figure 2A,B). A similar heterogeneity in PD1/PDL1 expression was observed in the 3 B-NHL samples analyzed with only 1/3 samples expressing either PD1 or PDL1 on >5% of neoplastic cells (data not shown).

These data suggest that a BCMA×PDL1 bsAb may be useful to target mBCMA-positive MM or B-NHL cells and hyperactivate the immune system within the tumor microenvironment.

### 3.2. Structure of the BCMA×PDL1 bsAb and Affinity Measurements

**Figure 3:**
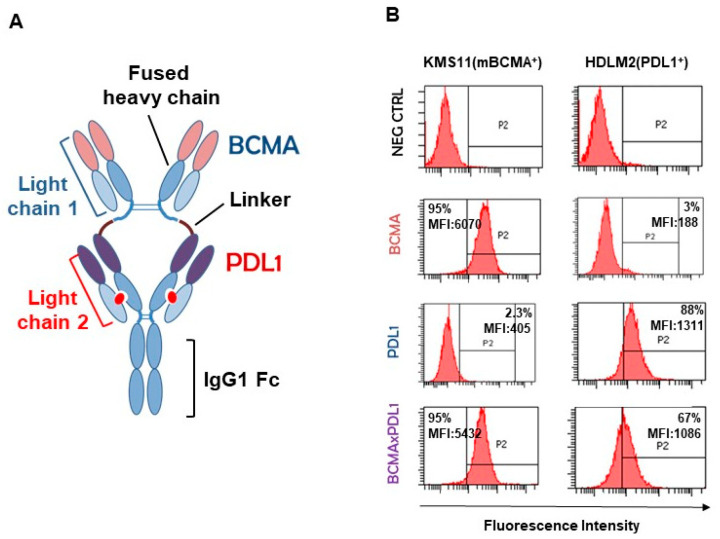
Structure and specificity of binding of chimeric BCMA×PDL1 bsAb. (**A**) Starting from the N-terminus, the fused heavy chain is composed of the anti-BCMA J22.9 antibody VH sequence-CH1 hinge1-A1linker-anti-PDL1 atezolizumab VH sequence-CH1 hinge2-CH2-CH3. The CH1 hinge-CH2 and CH3 sequences are from human IgG1. The two light chains (both k) are anti-BCMA J22.9 VL-CL and anti-PDL1 atezolizumab VL-CL. The red dot indicates the paired complementary mutations on CH1 and CL of the anti-PDL1 moiety to drive correct light chain pairing [6]. (**B**) Specificity of binding of the purified bsAb and respective mAbs was tested by flow cytometry on mBCMA^+^ and PDL1^+^ single-positive cell lines, KMS11 and HDLM2, respectively. MFI: mean fluorescence intensity. %: percentage of mBCMA-positive cells.

We designed and constructed a bsAb targeting both mBCMA and PDL1, based on our patented IgG1 structure, which is bivalent for each antigen and has a fully functional IgG1 Fc [6] (Figure 3A). As expected, the bsAb was able to specifically bind to both mBCMA and PDL1, as shown by the staining of cell lines expressing either mBCMA (KMS11) or PDL1 only (HDLM2) (Figure 3B).

**Figure 4:**
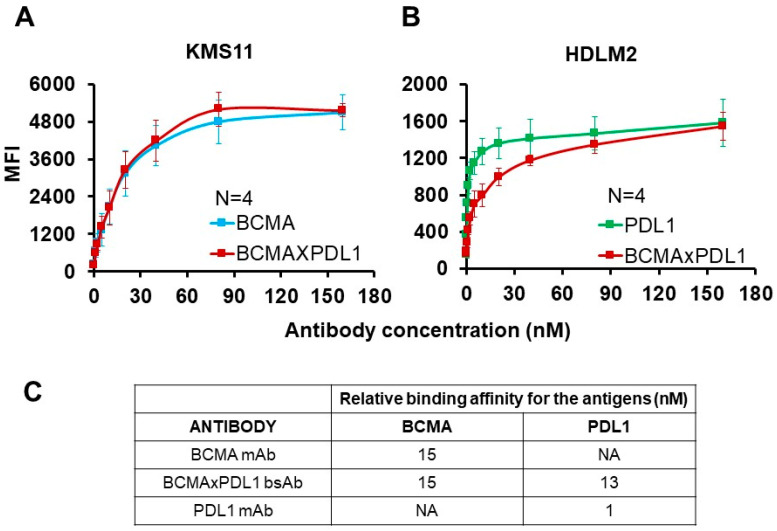
Relative binding affinity of BCMA×PDL1 bsAb and respective mAbs for target antigens. The relative affinity of the BCMA×PDL1 bsAb, anti-BCMA, and anti-PDL1 mAbs was tested by flow cytometry, using increasing concentrations of primary antibodies and detection with anti-human Fc-FITC secondary antibody. (**A**) Binding of bsAb and mAbs to mBCMA^+^ KMS11 cell line. (**B**) Binding of bsAb and mAbs to PDL1^+^ HDM2 cell line. (**C**) Relative binding affinities (IC_50_) for each antigen; NA: not applicable.

The binding affinity to the native antigens was also measured using the same cell lines stained with increasing amounts of the BCMA×PDL1 bsAb or parent mAbs. The binding affinity of bsAb was calculated to be about 15 nM for mBCMA, similar to the affinity of parent anti-BCMA mAb (Figure 4A,C). The affinity of bsAb for PDL1 was 13 nM, whereas it was about 1 nM for the parent anti-PDL1 mAb (Figure 4B,C). This is expected since the anti-PDL1 moiety is more internal to the molecule and may experience partial steric hindrance induced by the anti-BCMA moiety, reducing affinity ([6,14] and see below).

In order to further characterize the antigen binding properties of the bsAb and parent mAbs, we performed SPR assays, which allow the interaction between the antibodies and their targets to be studied in real-time and the binding constants (k_a_, k_d_, and K_D_ in Table 1) to be estimated. All the antibodies were immobilized in parallel chambers of the same chip and the soluble recBCMA and recPDL1 peptides were then injected, either alone or in succession.

**Figure 5:**
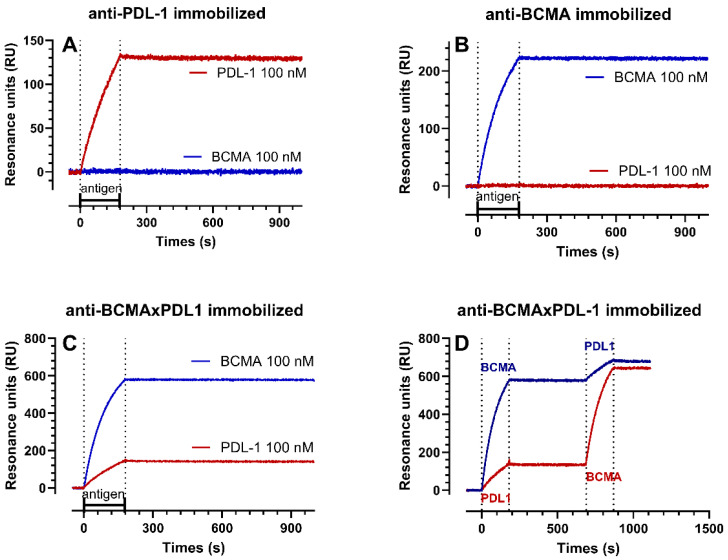
Surface plasmon resonance analysis. The sensorgrams shown were obtained by injecting recPDL1 or recBCMA, alone (**A**–**C**) or in succession (**D**) over immobilized anti-PDL1 (**A**), anti-BCMA (**B**), or BCMA×PDL1 bsAb (**C**,**D**). The antigens were flowed for 3 min, as indicated by the dashed lines.

Representative sensorgrams with the binding profiles are presented in Figure 5 and the binding constants are summarized in Table 1. No binding of recPDL1 was observed on immobilized anti-BCMA mAb and no binding of recBCMA was observed on immobilized anti-PDL1 mAb (Figure 5A,B), demonstrating specificity. In all the other cases, we observed very high-affinity interactions (sub-nanomolar K_D_ values) characterized by very fast association rate constants (k_a_) and very slow dissociation rate constants (k_d_) (Figure 5A–C and Table 1).

The main findings were that (1) recBCMA bound the bsAb with an affinity (mean K_D_ 19.5 pM) 5-fold higher than its affinity for the anti-BCMA mAb (K_D_ 106 pM). (2) recPDL1 bound the bsAb with an affinity (mean K_D_ 203 pM) 3-fold lower than its affinity for the anti-PDL1 mAb (K_D_ 71 pM). (3) The Rmax value (i.e., the maximum binding possible on the immobilized ligand) observed on the bsAb with recBCMA was higher than that with recPDL1 (691 vs. 241 RU on average). Taking into account the molecular weight of the proteins (52 kDa for recPDL1 and 32 kDa for recBCMA), we can calculate that the immobilized bsAb allows an occupancy of anti-BCMA arms about 5-fold greater than that of anti-PDL1. This is likely associated with the steric hindrance due to the disposition of the epitopes in the bsAb and is already identified with apparent decreased affinity to native PDL1 protein in the bsAb compared to mAb (Figure 3). (4) We also observed that if recPDL1 was injected after recBCMA (Figure 5D), its binding to bsAb decreased by 15–25%. This decrease can be due either to a decrease in the number of available sites (decrease in Rmax by 20% on average) or a 2-fold decrease in affinity, or both. This again is possibly due to the disposition of the epitopes in the bsAb, and/or to the conformational change induced by BCMA on the more internal PDL1 epitope (Figure 3). (5) When recBCMA was injected after recPDL1 (Figure 5D), a much smaller decrease in binding (7–12%) was observed.

We conclude that the internal anti-PDL1 moiety in the bsAb has a three-fold lower affinity for PDL1 in comparison to the mAb, and that this interaction is slightly reduced by previous BCMA binding. Moreover, the anti-PDL1 moiety in the bsAb appears 5-fold less accessible than the anti-BCMA moiety. Nonetheless, the overall affinity and binding to native PDL1 was in the sub-nM range, similar to that to BCMA, which should allow the blocking of PDL1 in the tumor microenvironment.

### 3.3. The BCMA×PDL1 bsAb Blocks PD1-PDL1 Interaction and APRIL Binding to BCMA

We next set out to verify that the bsAb could block the functions of its target antigens.

**Figure 6:**
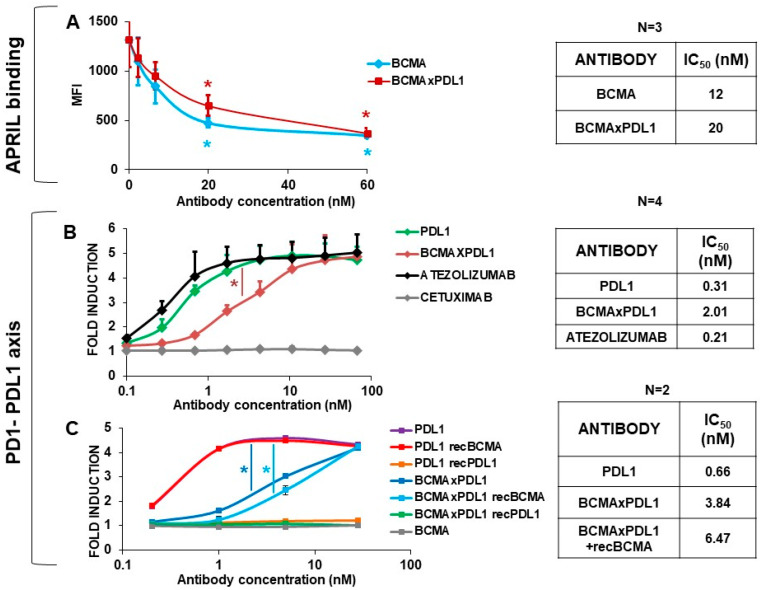
The BCMA×PDL1 bsAb blocks APRIL binding to mBCMA and PD1-PDL1 interaction. (**A**) To test the ability of bsAb to block APRIL binding to mBCMA, we used CEM-mBCMA^+^ cell line, increasing concentrations of bsAb and anti-BCMA mAb, a Flag-tagged APRIL protein, and an anti-Flag antibody. *: *p* < 0.05. (**B**) For assessing the inhibition of the PD1-PDL1 axis, we tested increasing concentrations of BCMA×PDL1 bsAb or anti-PDL1 mAb in the cell-based PD1/PDL1 Blockade Bioassay. (**C**) Cell-based PD1/PDL1 Blockade Bioassay in presence of recBCMA. Atezolizumab and cetuximab were used as positive and negative controls, respectively. *: *p* < 0.05 vs. PDL1.

APRIL (TNFSF13) is a trimeric molecule belonging to the tumor necrosis factor superfamily [23]. APRIL has been recognized as a proliferation ligand for BCMA and its interaction plays a critical role in the proliferation and survival of MM cells and normal PC [24,25,26,27]. APRIL also binds to heparan sulfate proteoglycans, such as syndecan 1, expressed on mature B cells [28]. In order to specifically measure APRIL binding to mBCMA without possible effects of syndecans, we used the T cell lines of CEM stably transfected with BCMA. The CEM-mBCMA^+^ cells were incubated with recombinant Flag-tagged APRIL protein, in the presence or absence of the anti-BCMA mAbs or bsAbs, and then probed with an anti-Flag antibody. As shown in Figure 6A, both anti-BCMA mAb and BCMA×PDL1 bsAb were able to block APRIL binding to mBCMA, with similar IC_50_ values of 12–20 nM. This suggests that the bsAb is able to block BCMA function.

We next tested the capacity of the PDL1 mAbs and bsAb to induce T cell activation, using a commercial TCR-mediated, luminescent T cell activation assay which is specifically blocked by the PDL1-PD1 interaction. As shown in Figure 6B, the anti-PDL1 mAb induced T cell activation with an efficacy similar to that of commercially available atezolizumab, with an IC_50_ of about 0.3 nM. The BCMA×PDL1 bsAb could also block the PD1-PDL1 inhibitory pathway and induce T cell activation, but at an 8–10-fold higher concentration (2 nM and *p* < 0.05) (Figure 6B). This is expected from the lower affinity/occupancy of the PDL1 moiety in the bsAb compared to mAb, as described above. Irrelevant control antibody cetuximab had no effect (Figure 6B).

We also investigated whether the occupancy of the anti-BCMA moiety of BCMA×PDL1 bsAb reduced PDL1 binding and functional efficacy, as suggested above by the SPR data. To do this, we added recBCMA to the antibody before addition to the T cells in the PD1/PDL1 Blockade Bioassay. As shown in Figure 6C, the addition of recBCMA reduced the T cell activation by about 15%, in line with the SPR data, suggesting a reduction in the capacity of BCMA×PDL1 to block PDL1-PD1 interaction in the presence of the BCMA target. The effect was not significant. Nonetheless, even with occupancy of the BCMA arm by excess recBCMA, the bsAb was able to block PDL1 and strongly induced T cell activation with an IC_50_ of about 8 nM.

We conclude that BCMA×PDL1 has the capacity to bind specifically to both targets with nM affinity and to effectively block the function of both mBCMA and PDL1 in vitro, also in the nM range. An inhibition of PDL1 function was observed even if the BCMA arm was occupied by BCMA.

### 3.4. BCMA×PDL1 Induces CDC

**Figure 7:**
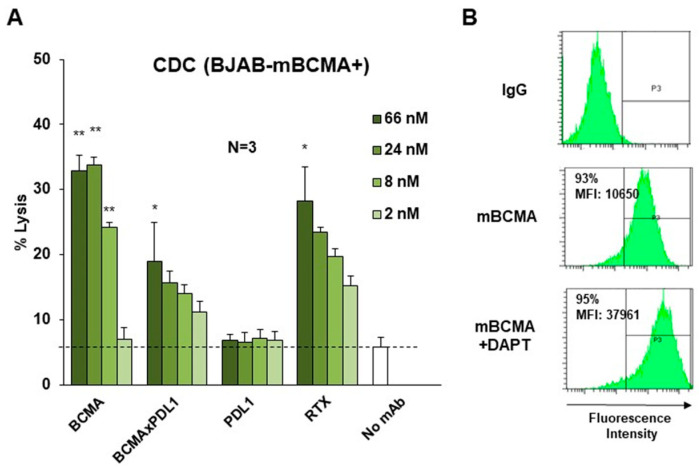
The BCMA×PDL1 bsAb mediates CDC of mBCMA^+^ cells. (**A**) The BJAB-mBCMA^+^ cell line was incubated with increasing concentrations of bsAb, mAbs, or RTX as positive control and in the presence of 50% HS as a source of complement. CDC was measured after 4 h by 7-AAD staining and flow cytometry. *: *p* < 0.05 and **: *p* < 0.01. (**B**) Flow cytometry histograms showing the expression of mBCMA of BJAB cells stably expressing BCMA in presence or absence of DAPT. MFI: mean fluorescence intensity. %: percentage of mBCMA-positive cells.

We next investigated whether the Fc portion of bsAb has functional activity. We first tested CDC using human serum as a source of complement and the BJAB cell line stably transduced with BCMA as a target. This cell line is also CD20^+^ and is a target of RTX, a strong CDC inducer. Anti-BCMA mAb induced 34% CDC at 24 nM, similar to what is observed with the gold-standard RTX antibody (Figure 7). BCMA×PDL1 bsAb showed 19% and 15% CDC at 66 nM and 24 nM, respectively (Figure 7). The functionality of bsAb was also tested in the absence of DAPT. In these conditions, CDC was not significant, presumably due to the low expression of BCMA without DAPT (Figure 7B and Supplementary Figure S2). Anti-PDL1 did not induce CDC even at high concentrations, as expected since the BJAB cell line is negative for PDL1 (data not shown).

The data demonstrate that BCMA×PDL1 bsAb can induce the CDC of appropriate targets. BsAb requires 3–10-fold higher concentrations than anti-BCMA mAb for similar efficacies. Nonetheless, CDC is induced by BCMA×PDL1 at optimal concentrations to similar levels as that triggered by RTX.

### 3.5. BCMA×PDL1 BsAb Induces ADCC by NK Cells

**Figure 8:**
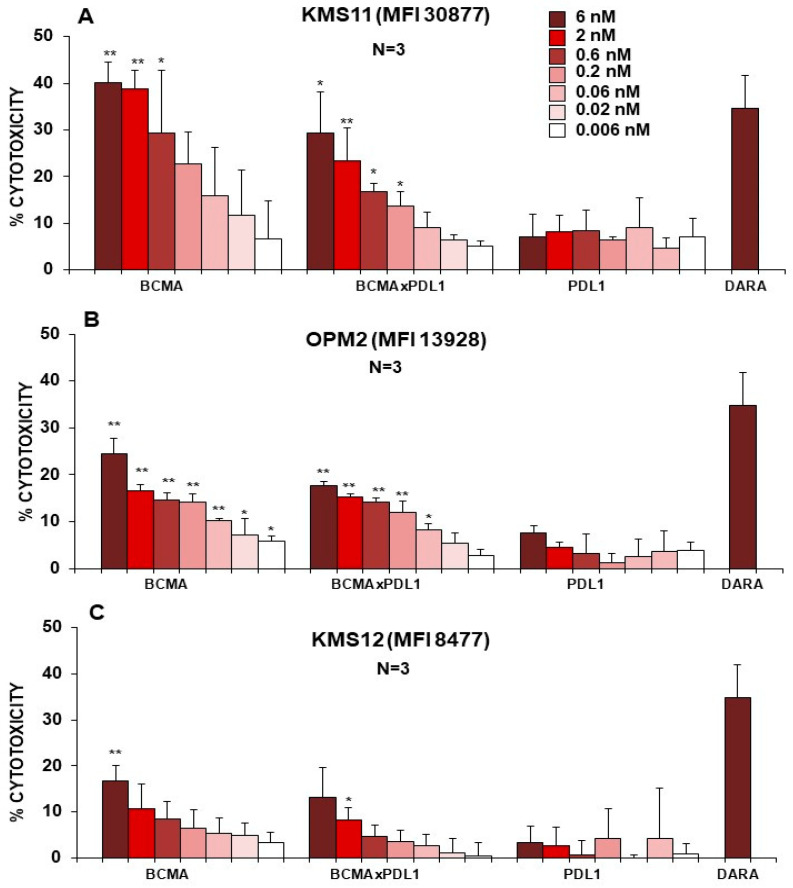
BCMA×PDL1 bsAb mediates ADCC (24 h killing assays). PBMCs were incubated with three different MM target cell lines (KMS11 (**A**), OPM2 (**B**) and KMS12 (**C**)) in the presence or absence of bsAb or control mAbs at the indicated concentrations. After 24 h, target cell death was measured using an Apoptosis/Necrosis detection kit and flow cytometry. *: *p* < 0.05 and **: *p* < 0.01. versus no mAb.

To probe the cell-mediated cytotoxic activity of the bsAb, we first performed standard ADCC assays, using PBMCs as a source of NK cells and three different MM cell lines as targets (KMS11, OPM2, KMS12), expressing different levels of mBCMA (MFI ranging from about 31,000 to 8500 following DAPT treatment). As shown in Figure 8, both anti-BCMA mAb and BCMA×PDL1 bsAb were able to induce cytotoxicity of the mBCMA^+++^ cell line and KMS11 cell line (MFI 30877) up to 40% and 30%, respectively. The bsAb showed the same efficacy as the BCMA mAb, but at a 3–10-fold higher concentration, presumably due to the conformation of the bsAb, with the Fc further distant from the antigen in the bsAb compared to the mAb format. Of note is that the efficacy of ADCC of the KMS11 cell line was similar to that observed with anti-CD38 mAb DARA used as the control. The ADCC of the OPM2 and KMS12 cells lines expressing lower levels of mBCMA (MFI 13,928 and 8477, respectively) was also observed with BCMA mAb and BCMA×PDL1 bsAb, albeit to a lower level (17–24% and 13–18%, respectively). As expected, anti-PDL1 mAb did not induce ADCC, as the three cell lines do not express this antigen (Figure 8 and Supplementary Figure S3). The fact that NK cells mediated cytotoxicity in this assay was confirmed by the induction of CD107a on NK cells in the presence, but not in the absence, of anti-BCMA mAb and BCMA×PDL1 bsAb (Supplementary Figure S4). The functionality of the bsAb and the parent anti-BCMA in inducing NK degranulation was weaker in the absence of DAPT, but still significant (Supplementary Figure S4B). Similar ADCC results were observed using an mBCMA^+^ B-NHL cell line as a target (PA698, Supplementary Figure S5).

### 3.6. Cytotoxicity Mediated by BCMA×PDL1 in 7-Day Killing Assays

**Figure 9:**
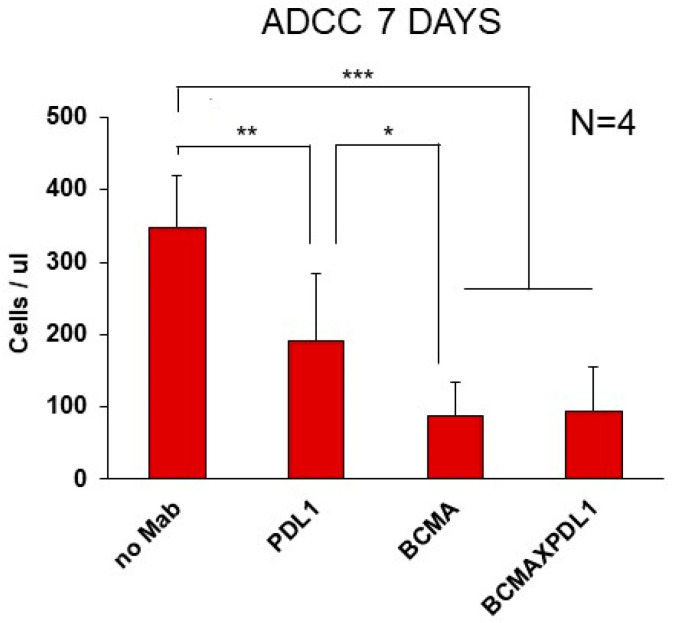
BCMA×PDL1 bsAb mediates ADCC in 7-day killing assays. PBMCs were co-cultured with KMS11 target cells and incubated with 6 nM of bsAb or mAbs. ADCC of target cells was assessed at 7 days by the absolute counting of live CD138^+^ KMS11 target cells by flow cytometry. *: *p* < 0.05, **: *p* < 0.01 and ***: *p* < 0.001.

In order to mimic a more physiological condition and also probe the effect of the combination of Fc and the anti-PDL1 moiety in mediating target cell killing, we performed cytotoxicity experiments over a 7-day period, using the KMS11 MM cell line as the target and PBMCs as effectors. We could indeed observe a strong cytotoxic effect of the BCMA mAb and BCMA×PDL1 bsAb at optimal concentrations (75% decrease in live cells by day 7). In contrast, anti-PDL1 mAb alone had a more limited, albeit significant, cytotoxic effect, with a 45% reduction in live cells at the same time point (Figure 9).

We conclude that the BCMA×PDL1 bsAb specifically kills mBCMA^+^ tumor cells in vitro in the presence of different immune effector cells.

## 4. Discussion

We report here the detailed in vitro characterization of a novel IgG1-like BCMA×PDL1 bsAb, both in terms of antigen binding and function. We show that the bsAb, which is bivalent for each antigen and has a fully functional Fc, binds specifically and with high affinity (nM range) to both targets. The affinity for both BCMA and PDL1 was similar, with an IC_50_ of about 15 nM in binding assays for the membrane-bound native antigens and a K_D_ of about 100 pM in SPR assays using recombinant protein fragments. The affinity for BCMA was similar, or even higher, in the bsAb and corresponding mAb, whereas the affinity for PDL1 apparently decreased about 3–10 fold in the bsAb compared to parent mAb. A similar decreased affinity of the internal moiety has previously been observed for other bsAbs designed on the same scaffold [6,14]. A more detailed SPR analysis suggested that this apparent reduction in affinity for the native protein was probably due to the more limited access of the internal Fabs to the target antigen by steric hindrance, i.e., a reduction in the maximal number of molecules able to bind to PDL1, rather than a decreased affinity per se. This is likely due to the more internal position of the anti-PDL1 moiety within the bsAb structure.

SPR assays investigating the binding to both antigens in succession demonstrated that the same bsAb molecule can bind both antigens simultaneously, even though a further small decrease in maximal binding to PDL1 was observed after the addition of recombinant BCMA. This is again probably due to the partial steric hindrance of the PDL1 moiety, that somewhat increased after BCMA binding, which may induce some conformational change to the molecule. In contrast, the bsAb bound BCMA with at least as high affinity as the parent mAb. The small difference (~20 vs. ~100 pM) may be due to the particular 3D conformation of anti-BCMA Fabs in bsAb compared to mAb.

We could also demonstrate that both bsAb and the anti-BCMA parent mAb specifically blocked APRIL binding to an mBCMA-positive cell line, with similar IC_50_, confirming the cell binding and SPR data. The inhibition of APRIL binding should also therefore inhibit NFkB activation which is induced by APRIL ([29] and IC, unpublished data) and supports mature B cell survival and proliferation. The block of APRIL adds an additional function to the bsAb, which may participate in tumor control.

BCMA×PDL1 bsAb could also functionally block the PD1-PDL1 axis and induce TCR-dependent T cell activation in vitro. We observed a good capacity of the bsAb to activate T cells, at a concentration about 10-fold higher than that required by anti-PDL1 mAb, as expected from the reduced binding to PDL1 of bsAb compared to parent mAb. Nonetheless, T cell activation was maximal using the antibody at 8 nM, even in the presence of the simultaneous binding of recombinant BCMA, which corresponds to a concentration of 2 μg/mL, a concentration that may be reached in vivo in the circulation as well as locally [30].

We could finally demonstrate that the Fc portion of bsAb is functional. Indeed, the BCMA mAb and BCMA×PDL1 bsAb were both able to induce the CDC of appropriate targets, as efficiently as gold-standard RTX, at least after mBCMA upregulation by gamma-secretase inhibitor. This is not surprising, since CDC requires a threshold level of target antigen [31]. It is foreseeable that a brief treatment with gamma-secretase inhibitors may be feasible in the clinic prior to anti-BCMA infusion, to boost the activity of the bsAb through CDC [32]. The BCMA×PDL1 bsAb also induced efficient ADCC in 24 h and 7-day killing assays, using PBMCs as effector cells. We could demonstrate NK activation and killing in 24 h assays, showing that this cell type was indeed activated by the bsAb IgG1 Fc. In the 7-day killing assays, we could demonstrate the greater efficacy of BCMA×PDL1 compared to anti-PDL1 alone.

Altogether, these in vitro studies characterize quite in detail the binding characteristics and function of the new BCMA×PDL1 bsAb in vitro, compared to the parent mAbs. The aim of this work was to design a therapeutic bsAb that would be directed to the tumor, through its anti-tumor-associated antigen moiety (TAA, anti-BCMA), and then induce immune cell activation and killing in the microenvironment, by blocking in situ the PD1-PDL1 axis. Such blocking of PD1-PDL1 signaling may be more effective because it would take place locally, close to the tumor site. This may also avoid the non-specific and toxic effect that is often observed with anti-PDL1/PD1 mAbs systemically. In our view, such a molecule should have an affinity for the TAA at least as high as that to the ICI, in order to optimally achieve this goal. This was the case for our bsAb, which showed similar affinity for both antigens, in conditions of binding to a single antigen or to both simultaneously. Furthermore, the affinity and functional activity of the bsAb in the nM range demonstrated here suggest that an effective concentration of bsAb may be achieved in vivo. The bsAb was indeed able to induce significant CDC, ADCC, and target cell killing in the presence of different immune cells. One possible disadvantage of the present molecule is the partial steric hindrance that was observed for PDL1 binding, which may reduce the efficacy of the anti-ICI moiety. We also tested a different molecule with a more flexible and slightly longer linker, downstream of the first hinge (called GS, carrying 12 amino acids instead of 11 for the semi-rigid A1 linker) [6], but the behavior of this molecule was very similar to the one presented here (JG and IC, unpublished data). On the basis of the detailed analyses shown here, future work may investigate other formats of the bsAb with still longer flexible linkers, in order to reduce steric hindrance, but such molecules should also foresee to select appropriate relative affinities of the two moieties.

The analyses of BCMA and PD1/PDL1 expression performed here confirmed that anti-BCMA mAbs and BCMA×PDL1 bsAbs may be useful therapeutic drugs not only for MM but also for a proportion of more differentiated B-NHL, which in some cases express BCMA to a similar degree as MM cells, at least after the inhibition of ɣ-secretase. This is in agreement with previous data on BCMA expression in MM and B-NHL [7,8,22]. We also analyzed PD1 and PDL1 expression in MM and B-NHL primary BM or PB samples and demonstrated that one of these molecules is frequently expressed either on the tumor cells or on immune cells (T lymphocytes/monocytes/macrophages) in the tumor microenvironment, suggesting that BCMA×PDL1 may indeed be a useful therapeutic tool in both MM and B-NHL [33,34].

Other bsAbs-targeting checkpoint inhibitors have reached the clinic and have shown in some cases very promising activities [35]. Most of these bsAbs target two different ICIs or an ICI and an immune activator. Only a few have been designed to target a TAA as well as an ICI. The latter include EGFR×PD1, HER2×PD1 [36], and PDL1×EGFR [37]. Also, molecules targeting TGFβ and PDL1 have shown promising activity in clinical studies [38].

Clearly, the next step will involve the production of the bsAb in mammalian cells and its testing in immunocompetent or humanized models in vivo, which are suitable for investigating the therapeutic activity of anti-ICI also targeting a human TAA [39,40]. Such a model could include the generation of murine lymphoma or myeloma cell lines, stably transfected with human BCMA, to be used in syngeneic immunocompetent mice, similar to what has been performed for anti-CD20 antibodies, given that the anti-PDL1 moiety of the bsAb also recognizes murine PDL1 [41,42].

## Figures and Tables

**Table 1:** Binding constants determined by SPR studies.

	k_a_	k_d_	K_D_	Rmax
	1/Ms	1/s	nM	RU
recPDL1 on PDL1 mAb	4.9–5.5 × 10^4^	1.1–5.9 × 10^−6^	0.02–0.12	222–227
recPDL1 on BCMA×PDL1 bsAb	3.8–6.2 × 10^4^	0.4–1.8 × 10^−5^	0.11–0.29	212–271
recBCMA on BCMA mAB	5.9–8.1 × 10^4^	5.9–8.6 × 10^−6^	0.11–0.10	289–312
recBCMA on BCMA×PDL1 bsAb	1.0–1.0 × 10^5^	1.3–2.7 × 10^−6^	0.01–0.03	683–700

The sensorgrams were fitted with a 1:1 Langmuir equation to obtain association and dissociation rate constants (k_a_ and k_d_), from which K_D_ is calculated (k_d_/k_a_), and Rmax values, i.e., the maximum possible signal (in RU). The results of two independent experiments are shown.

## Data Availability

Data are contained within the article and Supplementary Materials.

## Supplementary Materials

The following supporting information can be downloaded at https://www.mdpi.com/article/10.3390/antib13010015/s1, Figure S1: Expression of mBCMA in MM and B-NHL patients; Figure S2: CDC of BCMA×PDL1 bsAb in absence of the ɣ.-secretase inhibitor DAPT; Figure S3: PDL1 expression in MM cell lines; Figure S4: BCMA×PDL1 bsAb activates NK cells; Figure S5: BCMA×PDL1 bsAb mediates ADCC in a mBCMA+ B-NHL cell line.
