# Supplementary material for: Development of a Bispecific IgG1 Antibody Targeting BCMA and PDL1
Source: Antibodies (Basel). 2024 Feb 20;13(1):15. doi: 10.3390/antib13010015 (PMC10885062; doi:10.3390/antib13010015)

Figure S1: Expression of mBCMA in MM and B-NHL patients.

mBCMA expression in presence or absence of ɣ-secretase inhibitor DAPT in MM and B-NHL neoplastic cells from 23 patients. The expression was assessed by flow cytometry. (A) Percentage of mBCMA+ cells. (B) Mean fluorescence intensity, MFI. On the x-axis patients are identified with arabic numerals from 1 to 23. Patients 22 and 23 were diagnosed with marginal zone lymphoma (MZL)

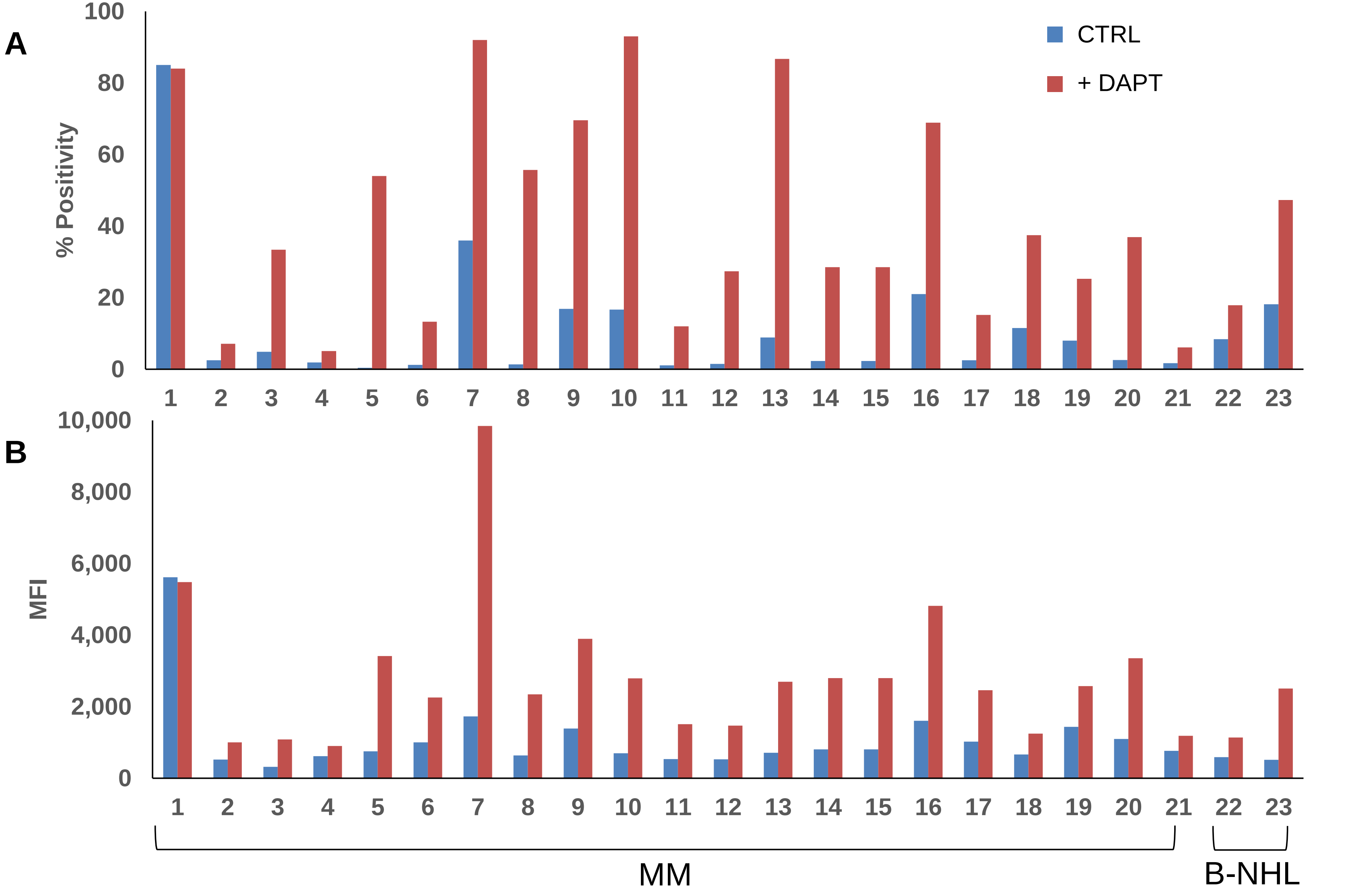

Supplement: Supplementary file 1 [file antibodies-13-00015-s001.zip › FigureS1 rev proofs.pdf]
