# Supplementary material for: Development of a Bispecific IgG1 Antibody Targeting BCMA and PDL1
Source: Antibodies (Basel). 2024 Feb 20;13(1):15. doi: 10.3390/antib13010015 (PMC10885062; doi:10.3390/antib13010015)

Figure S2: CDC of BCMAxPDL1 bsAb in absence of the ɣ.-secretase inhibitor DAPT.

The BJAB-mBCMA+ cell line was incubated with increasing concentrations of bsAb, mAbs or rituximab (RTX) as positive control and in the presence of 50% HS as a source of complement. BJAB-mBCMA+ cell line was not incubated overnight with DAPT before the experiments. CDC was measured after 4 h by 7-AAD staining and flow cytometry. *:p≤0.05 vs No mAb.

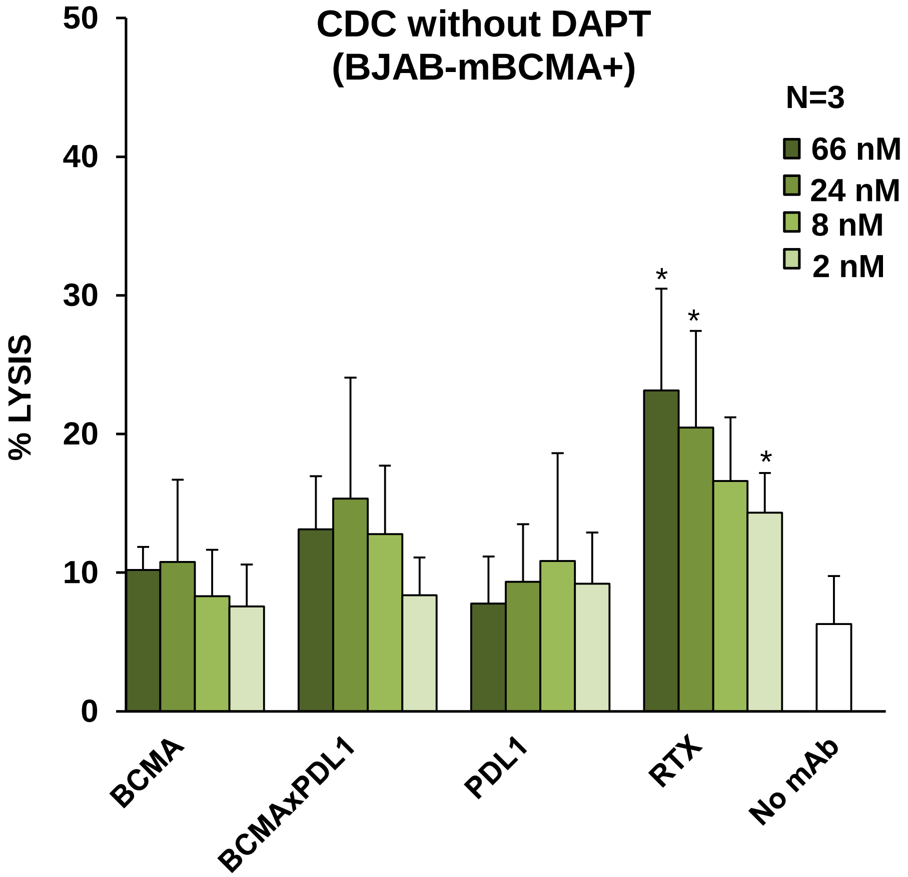

Supplement: Supplementary file 1 [file antibodies-13-00015-s001.zip › FigureS2.pdf]
