# Supplementary material for: Development of a Bispecific IgG1 Antibody Targeting BCMA and PDL1
Source: Antibodies (Basel). 2024 Feb 20;13(1):15. doi: 10.3390/antib13010015 (PMC10885062; doi:10.3390/antib13010015)

Figure S3: PDL1 expression in MM cell lines.
PDL1 expression was analysed by flow cytometry on human MM cell lines: KMS11, OPM2 and KMS12. Cells were stained with an anti-human PDL1-PE (clone MIH1) or with the respective isotype control. All cell lines did not express PDL1.

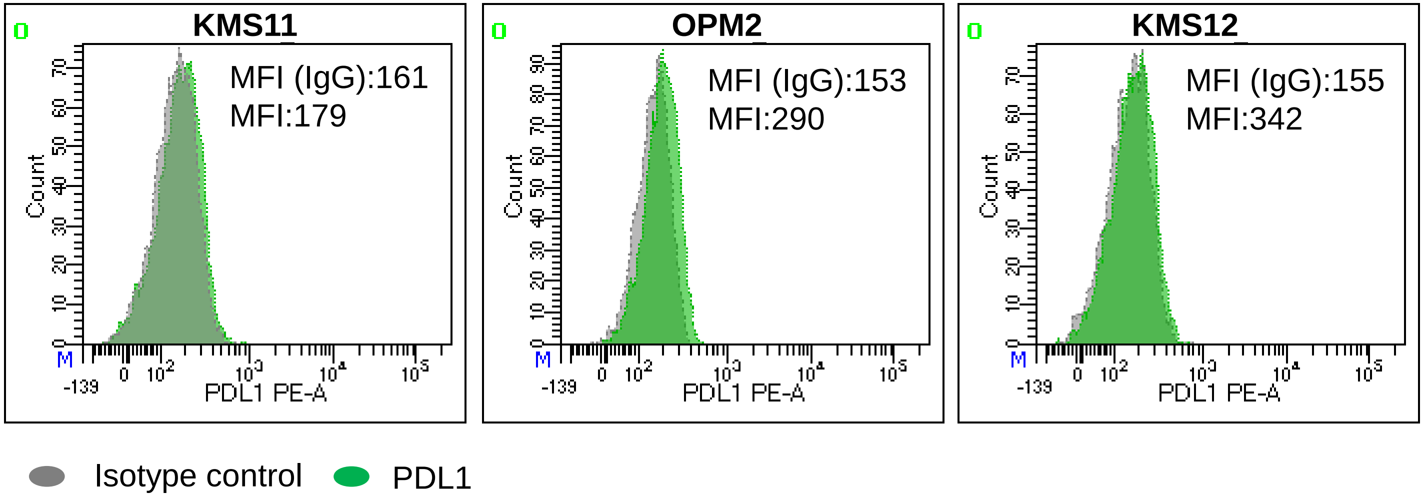

Supplement: Supplementary file 1 [file antibodies-13-00015-s001.zip › FigureS3.pdf]
