# Supplementary material for: Development of a Bispecific IgG1 Antibody Targeting BCMA and PDL1
Source: Antibodies (Basel). 2024 Feb 20;13(1):15. doi: 10.3390/antib13010015 (PMC10885062; doi:10.3390/antib13010015)

Figure S4: BCMAxPDL1 bsAb activates NK cells.

PBMCs as source of NK cells were incubated with KMS11 cells in presence or absence of bsAb or control mAbs at the indicated concentrations. NK cell activation was measured as the percentage of CD107a on $CD56^{+}$ cells by flow cytometry. Panel A and B show two different experimental conditions: KMS11 cells treated with 1 µM DAPT (A) or maintained in standard culture medium (B) overnight before experiments. DARA: daratumumab; *p<0.05, **p<0.01 and ***p<0.01 versus no mAb.

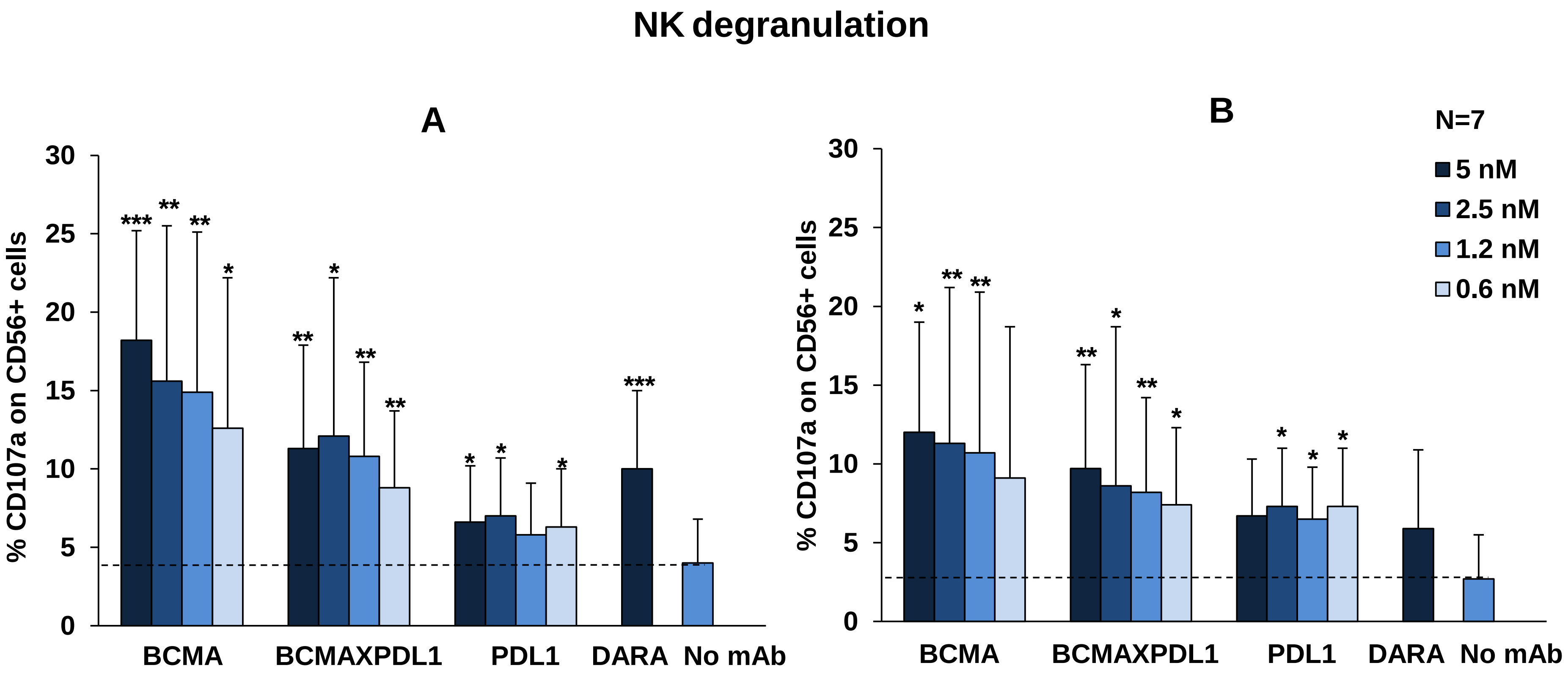

Supplement: Supplementary file 1 [file antibodies-13-00015-s001.zip › FigureS4.pdf]
