# Supplementary material for: Development of a Bispecific IgG1 Antibody Targeting BCMA and PDL1
Source: Antibodies (Basel). 2024 Feb 20;13(1):15. doi: 10.3390/antib13010015 (PMC10885062; doi:10.3390/antib13010015)

Figure S5: BCMAxPDL1 bsAb mediates ADCC in a mBCMA+ B-NHL cell line.
PBMCs were incubated with the PA698 cell line in presence or absence of bsAb or control mAbs at the indicated concentrations. After 24 hours, target cell death was measured using an Apoptosis/Necrosis detection kit and flow cytometry. DARA: daratumumab. *:p<0.05 versus no mAb.

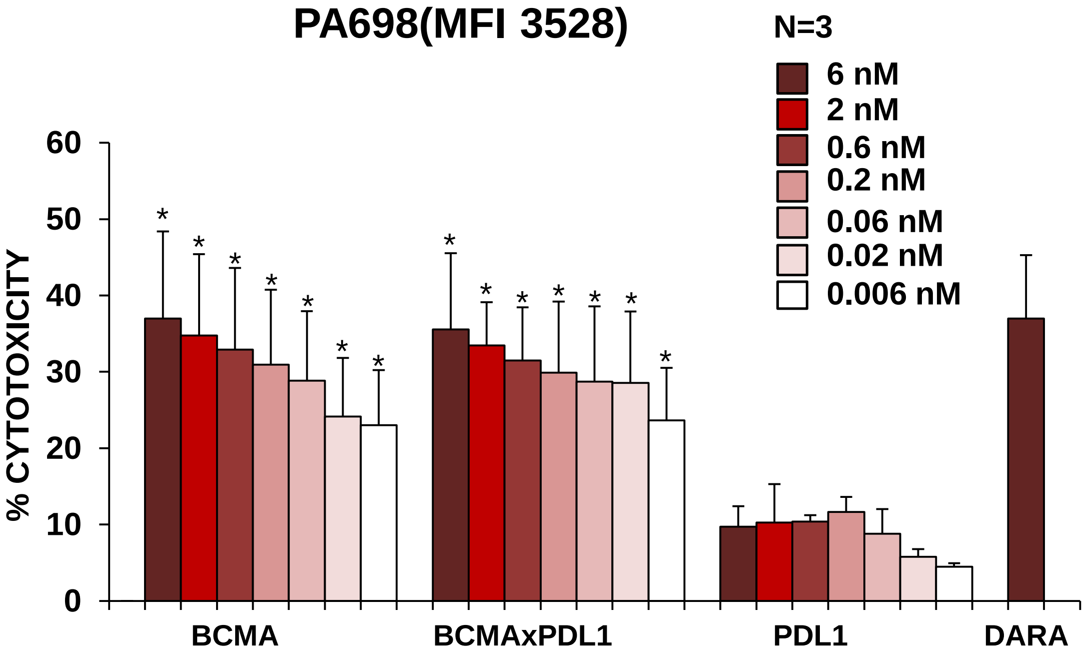

Supplement: Supplementary file 1 [file antibodies-13-00015-s001.zip › FigureS5 rev proofs.pdf]
